# Smart goggles augmented reality CT–US fusion compared to conventional fusion navigation for percutaneous needle insertion

**DOI:** 10.1007/s11548-024-03148-5

**Published:** 2024-05-30

**Authors:** Tabea Borde, Laetitia Saccenti, Ming Li, Nicole A. Varble, Lindsey A. Hazen, Michael T. Kassin, Ifechi N. Ukeh, Keith M. Horton, Jose F. Delgado, Charles Martin, Sheng Xu, William F. Pritchard, John W. Karanian, Bradford J. Wood

**Affiliations:** 1 Center for Interventional Oncology, Radiology and Imaging Sciences, Clinical Center, National Institutes of Health, 10 Center Drive, Room 3N320, MSC 1182, Bethesda, MD 20892 USA; 2 Henri Mondor Biomedical Research Institute, Inserm U955, Team N°18, Créteil, France; 3 Philips Healthcare, Cambridge, MA 02141 USA; 4 Department of Radiology, Georgetown Medical School, Medstar Washington Hospital Center, Washington, DC 20007 USA; 5 Fischell Department of Bioengineering, University of Maryland, College Park, MD 20742 USA; 6 Department of Interventional Radiology, Cleveland Clinic, Cleveland, OH 44195 USA

**Keywords:** Augmented reality, Radiology, Interventional, Image-guided biopsy, Biopsy, Needle

## Abstract

**Purpose:**

Targeting accuracy determines outcomes for percutaneous needle interventions. Augmented reality (AR) in IR may improve procedural guidance and facilitate access to complex locations. This study aimed to evaluate percutaneous needle placement accuracy using a goggle-based AR system compared to an ultrasound (US)-based fusion navigation system.

**Methods:**

Six interventional radiologists performed 24 independent needle placements in an anthropomorphic phantom (CIRS 057A) in four needle guidance cohorts (*n* = 6 each): (1) US-based fusion, (2) goggle-based AR with stereoscopically projected anatomy (AR-overlay), (3) goggle AR without the projection (AR-plain), and (4) CT-guided freehand. US-based fusion included US/CT registration with electromagnetic (EM) needle, transducer, and patient tracking. For AR-overlay, US, EM-tracked needle, stereoscopic anatomical structures and targets were superimposed over the phantom. Needle placement accuracy (distance from needle tip to target center), placement time (from skin puncture to final position), and procedure time (time to completion) were measured.

**Results:**

Mean needle placement accuracy using US-based fusion, AR-overlay, AR-plain, and freehand was 4.5 ± 1.7 mm, 7.0 ± 4.7 mm, 4.7 ± 1.7 mm, and 9.2 ± 5.8 mm, respectively. AR-plain demonstrated comparable accuracy to US-based fusion (*p* = 0.7) and AR-overlay (*p* = 0.06). Excluding two outliers, AR-overlay accuracy became 5.9 ± 2.6 mm. US-based fusion had the highest mean placement time (44.3 ± 27.7 s) compared to all navigation cohorts (*p* < 0.001). Longest procedure times were recorded with AR-overlay (34 ± 10.2 min) compared to AR-plain (22.7 ± 8.6 min, *p* = 0.09), US-based fusion (19.5 ± 5.6 min, *p* = 0.02), and freehand (14.8 ± 1.6 min, *p* = 0.002).

**Conclusion:**

Goggle-based AR showed no difference in needle placement accuracy compared to the commercially available US-based fusion navigation platform. Differences in accuracy and procedure times were apparent with different display modes (with/without stereoscopic projections). The AR-based projection of the US and needle trajectory over the body may be a helpful tool to enhance visuospatial orientation. Thus, this study refines the potential role of AR for needle placements, which may serve as a catalyst for informed implementation of AR techniques in IR.

**Supplementary Information:**

The online version contains supplementary material available at 10.1007/s11548-024-03148-5.

## Introduction

Precision and targeting accuracy are essential for successful image-guided percutaneous needle interventions. A precise needle insertion is in part dependent on careful treatment planning and reliable intraprocedural image guidance. Needle navigation technologies may be helpful for composite ablations, ultrasound, unenhanced CT or CBCT-invisible targets, for high risk procedures, or for inexperience or uncertainty of the operator. In recent years, a range of image-guided needle navigation and automation systems based on electromagnetic (EM) or optical devices, laser systems, and robotics [1–5] have been developed. These have been deployed in clinic with the purpose of increasing accuracy, reliability, standardization, and reproducibility, as well as reducing radiation dose and variability among operators. Image-fusion platforms merging the benefits of conventional imaging modalities such as US, CT, PET, and MRI have been incorporated into clinical practice [6]. Commercially available ultrasound-based fusion systems enable preprocedural treatment planning by generating target-specific needle trajectories and live orientation (i.e., in CT space) and try to account for intraprocedural respiratory motion with EM tracking or gating. With fusion navigation, the operator’s attention still alternates between the monitor and the patient, requiring experience in 3D spatial perception of anatomy and inter-related 2D navigational planes.

The emergence of augmented reality (AR) as a navigational tool in interventional radiology provides additional information by superimposing three-dimensional, holographic or stereoscopic displays over the procedural environment [7–9]. Recently, AR has found its way into interventional oncology as a direct guidance modality for percutaneous biopsies and ablations with the capacity to facilitate visuospatial orientation and hand–eye coordination as well as to provide swift access to real-time patient information. AR has the potential to improve standardization and reproducibility, reduce inter-user variability, and shorten learning curves [7, 10–12]. A current goggle-based AR system provides ultrasound-CT fused AR on a single display, which has not been specifically reported in recent studies of AR in IR [13, 14].

This study aims to evaluate needle placement accuracy of this goggle-based augmented reality guidance system with and without holographic-like, stereoscopic projections compared to a commercial ultrasound-based navigation system in a phantom model.

## Materials and methods

### Phantom model

An anthropomorphic abdominal phantom (Model 057A, CIRS Inc., Norfolk, VA) with pre-existing, CT- and US-visible, target lesions was used. Six lesions (liver: *n* = 5, kidney *n* = 1) with diameters ranging from 6.5 to 15.0 mm were targeted. The corresponding skin entry points were pre-determined resulting in a variety of insertion depths (mean needle depth 7.2 ± 1.6 cm, range 4.9–9.3 cm) and needle angulations. Six 2-mm metal spheres were placed on the phantom surface to identify needle entry points on preprocedural CT imaging. The entry points were paired with targets to force out-of-plane needle insertion when using ultrasound. The six paired targets and skin insertion sites were identical for all operators and insertion techniques.

### Needle insertion methods and preprocedural planning

#### Ultrasound-based fusion system

**Fig. 1:**
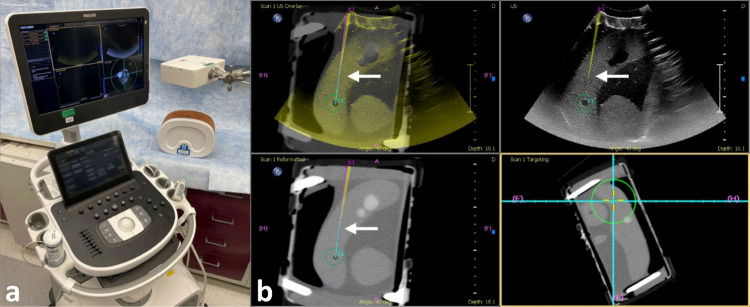
US-based fusion navigation system with EM-tracked needle and US transducer. **a** Ultrasound-based fusion device consisting of an ultrasound system, electromagnetic field generator and patient tracker. **b** Tracking display with blended fusion image of ultrasound and CT (top left), orthogonal views that include the tracked needle (top right, bottom left), and a bull’s-eye view along the axis of the tracked needle with the location of the needle centered in the yellow cross and the target centered in the teal lines (bottom right). The purple line is the planned needle path for the paired entry point and target, labeled E1 and T1, respectively. The yellow line is the tracked needle location with the dotted line showing the forward trajectory (yellow dots proximal to the target and teal dots distal to the target). The tip of the needle based on tracked location is indicated by the white arrow. The green circles in the fusion and the two orthogonal views appear if the target is in the image. The radius of the green circle in the bull’s-eye view represents the distance from the needle tip to the plane of the target

The ultrasound-based fusion system (PercuNav™, Philips, Bothell, WA) is a commercially available navigation system that enables multi-modality image fusion and needle navigation with tracked ultrasound. The system consists of an US workstation with a guidance tracking system, an EM field generator, and an EM-tracked US probe, patient reference, and needle (Fig. 1a). A preprocedural CT (Brilliance MX8000 IDT 16-section Detector CT, Philips, Cleveland, OH) of the phantom was performed (120 kVp, 300 mA, collimation 16 × 1.75, reconstructed as 2-mm slices at 1.5 mm intervals) and uploaded to the platform. The paired targets (*n* = 6) and skin entry points were identified on the preprocedural CT, and the needle trajectories were planned. The US was registered to the preprocedural CT based on three to five matched internal anatomic landmarks. The targets, skin entry points, and needle trajectories were displayed on the fused US and CT image (Fig. 1b). A 16-gauge, 11-cm EM-tracked needle was used (Coaxial Needle Tracker, Philips PercuNav™, Bothell, WA). Following target and entry point selection, the tracked needle was advanced by centering the needle, skin entry point, and target on a center cross in the bull’s-eye view along the axis of the needle. The size of a circle surrounding the crosshairs represented the depth of the needle tip in relation to the plane of the target (Fig. 1b). Users were directed to navigate based upon the bull’s-eye view.

### Goggle-based AR guidance system

**Fig. 2:**
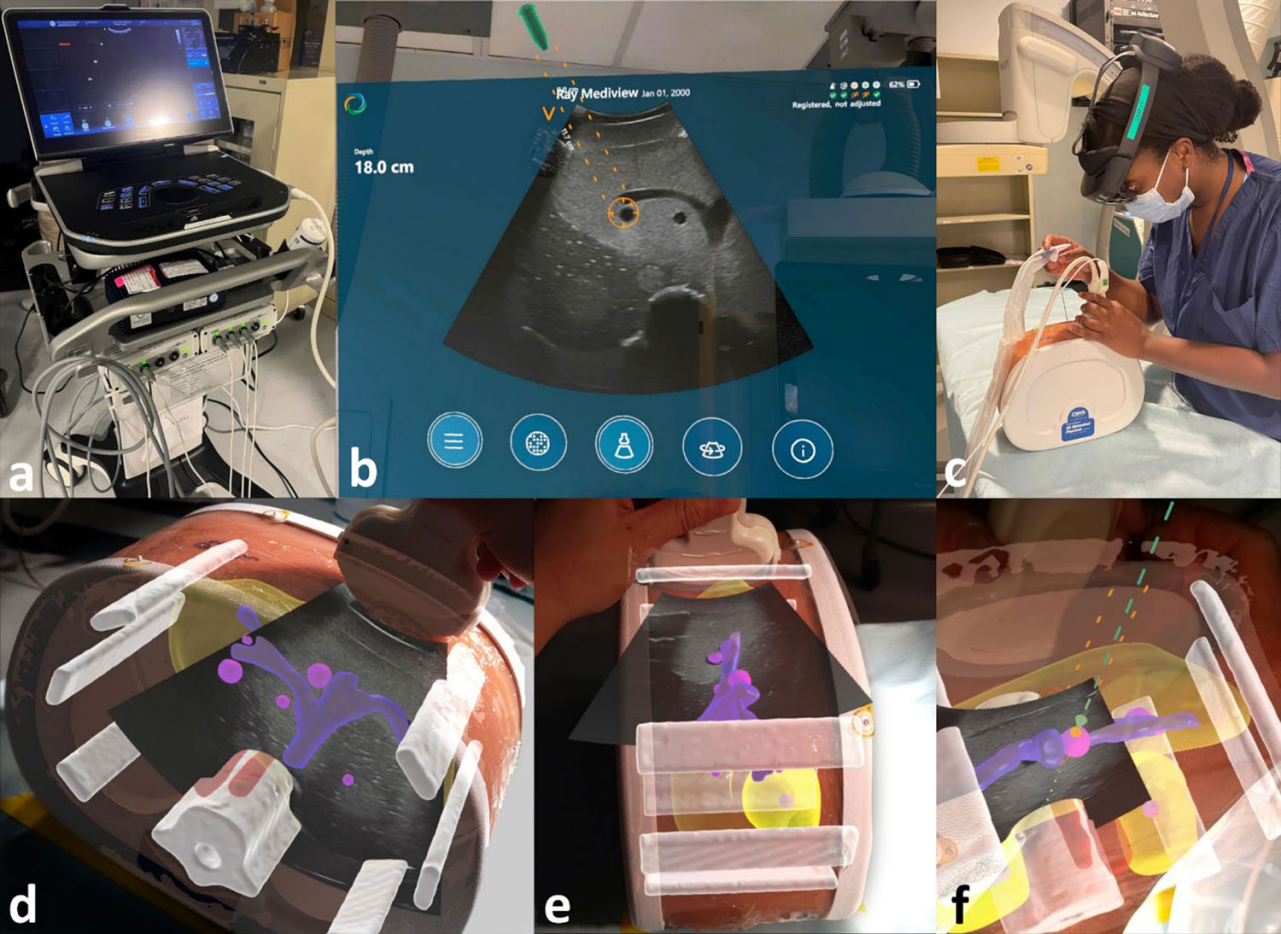
Goggle-based augmented reality guidance system overview. **a** Augmented reality device. **b** Off-patient display, an interactive user interface which also displays the real-time ultrasound and needle guide (orange dotted lines). **c** Operation of the system using smart goggles and an electromagnetically tracked needle and transducer. **d–f** Phantom body views with projection of real-time ultrasound and preprocedurally segmented CT-based stereoscopically projected anatomy: bones (white), skin (transparent orange), liver (yellow), portal vein (purple), target lesions (magenta). **f** Additional depiction of electromagnetically tracked needle path (green dotted line) inside the given, projected needle guide territory (orange dotted lines)

The goggle-based system (XR90TM, MediView XR, Inc., Cleveland, OH) is an AR-based medical device that stereoscopically projects and fuses US with a 3D anatomical model based on CT imaging to form a virtual, holographic-like 3D image in real space (Fig. 2). The system is comprised of commercially available smart goggles (HoloLens 2, Microsoft, Redmond, WA) which interface with an US system (Vivid iq, GE Healthcare, Chicago, IL) and an EM field generator positioned below the table (Northern Digital, Waterloo, Canada). Fiducial spot markers (Beekley Medical, Bristol, CT) were placed onto the skin surface of the phantom for segmentation. A preprocedural thin-sliced CT of the phantom was acquired to segment anatomical structures and targets for a detailed stereoscopic projection (120 kVp, 300 mA, collimation 16 × 0.75, reconstructed as 1.0 mm slices at 0.5 mm intervals). Segmentation was performed using open-source software (3D Slicer, URL https://www.slicer.org/) [15]. Registration markers with both optical image targets and EM sensors (MediView XR, Inc.) were placed on top of the prior CT spot markers to allow for simultaneous optical and EM registration. Eye calibration of the smart goggle was performed by each operator prior to the procedure. A 16-gauge, 17.7-cm EM-tracked needle was used (eTRAX, Civco, Kalona, IA). After combined registration of skin fiducials and EM tracking of the US probe and tracked needle, the 3D stereoscopically projected anatomy and real-time US images were superimposed over the phantom body. 3D anatomic structures could be hidden from view by voice command providing only the real-time US and needle navigation on the phantom body. Each target was manually selected by a finger pointing motion on the off-patient display (Fig. 2b). Following target selection, the EM-tracked needle was inserted and slowly advanced by aligning the projected needle tip with the selected target (Fig. 2c–f).

### Freehand

For freehand needle insertions, operators individually planned needle trajectories and depth for each target on preprocedural CT phantom images. Interval CT imaging during needle placement was not permitted in order to equalize radiation exposure among cohorts. Needle placement was performed cognitively as a single pass without retraction or repositioning.

### Phantom procedure

Six interventional radiologists with varied clinical experience (≤ 2 years, *n* = 2; 5–10 years, *n* = 2; and > 20 years, *n* = 2) each targeted all six pre-determined targets in four cohorts: US-based fusion (*n* = 6), goggle-based AR with CT-based stereoscopically projected anatomy of organs, skeletal structures, skin, and targets (AR-overlay, *n* = 6), goggle-based AR without stereoscopic projections (AR-plain, *n* = 6), and freehand (*n* = 6) for a total of 24 insertions per operator. The order of target insertions within each cohort was randomized among operators. All operators were instructed to insert the needle into the center of each spherical target, with a single placement attempt. Only forward motion was allowed; retracting or repositioning the needle was not permitted. Each operator performed three consecutive needle placements after which a CBCT scan (Allura Xper FD20 X-ray System, Philips, Best, The Netherlands) was acquired for measurement of placement accuracy, followed by removal of the three needles and insertion of the three remaining targets. All operators were blinded to the CBCT images. The primary endpoints were placement accuracy (defined as the distance between the needle tip and the center of the spherical target), placement time (measured from the initial skin puncture until operator satisfaction with final position), and procedure time (measured from start of trajectory planning of the first target to end of placement of the last of the six needles). Secondary endpoints were system preparation time (time from turning on system to time of registration), planning time (for US-based fusion: time of selecting targets and entry points; for goggle AR and freehand: time from selecting a target to skin puncture), registration time (time for registration process), and training time (time to independently use system).

### Placement accuracy

Placement accuracy was determined using 3D Slicer (URL https://www.slicer.org/) [15]. The *x*, *y*, and *z* coordinates of each target center and needle tip were recorded. The Euclidean distance between the target center and the needle tip was calculated using the Pythagorean theorem:

$${\text{distance}} \left({\text{mm}}\right)= \sqrt{{({x}_{1}-{x}_{2}\text{ )}}^{2}+ {({y}_{1}-{y}_{2})}^{2}+{({z}_{1}-{z}_{2})}^{2}}$$

### Statistical analysis

All statistical tests were computed in the open-source software R (R Foundation for Statistical Computing, Vienna, Austria. URL https://www.R-project.org/). The Shapiro–Wilk normality test revealed a non-Gaussian distribution of the nominal variables of primary endpoints including placement accuracy, placement time and procedure time. Descriptive statistics were presented as mean ± SD. The Kruskal–Wallis test was performed to compare operators (*n* = 6) and study cohorts (*n* = 4) reported with *p*-values. A post hoc Dunn’s test was used to adjust the *p*-values in multiple comparisons using Bonferroni–Holm correction criteria in non-Gaussian distributed variables. The Tukey fence method was used to detect outliers [16].

## Results

### Placement accuracy

**Fig. 3:**
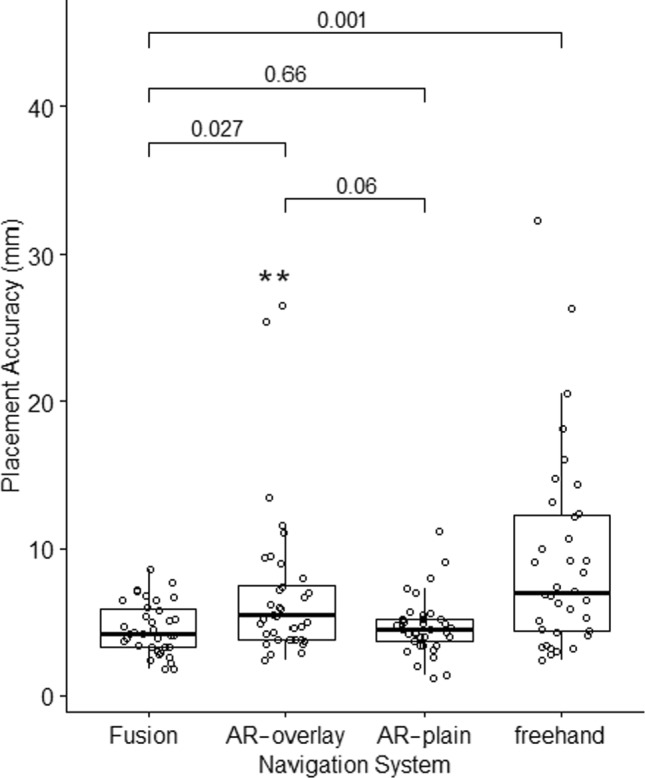
Needle placement accuracy. Needle placement accuracy, pooled for all six operators in each cohort, show greater variability with freehand compared to navigation technologies. Fusion = ultrasound-based fusion, AR-overlay = goggle-based augmented reality system with stereoscopically projected anatomy, AR-plain = goggle-based augmented reality system without anatomical projections, Freehand = single, cognitive needle insertion without navigation system. *Data points from two different operators that represent outliers but are included here

Mean needle placement accuracy using ultrasound-based fusion, goggle-based AR with (AR-overlay) and without (AR-plain) stereoscopically projected anatomy, and freehand was 4.5 ± 1.7 mm, 7.0 ± 4.7 mm, 4.7 ± 1.7 mm, and 9.2 ± 5.8 mm, respectively (Fig. 3, Table 1). Post hoc Bonferroni–Holm correction for multiple comparisons demonstrated no difference in needle placement accuracy for US-based fusion and AR-plain (*p* = 0.66). Placement accuracy of AR-overlay compared to AR-plain was *p* = 0.06. US-based fusion compared to AR-overlay was *p* = 0.03. US-based fusion and AR-plain showed greater placement accuracy compared to freehand (*p* = 0.001 and *p* = 0.002, respectively). Freehand and AR-overlay showed similar needle placement accuracy (*p* = 0.24). Application of the Tukey fence method identified a single data point from each of two different operators that fell outside the upper outer fence. Both had been noted as having technical or operator error in registration. With exclusion of these points, mean placement accuracy for the AR-overlay cohort became 5.9 ± 2.6 mm. Following post hoc Bonferroni–Holm correction, needle placement accuracy of the modified AR-overlay cohort compared to US-based fusion and AR-plain were *p* = 0.07 and *p* = 0.12, respectively. There was no difference in placement accuracy between operators with ≤ 2 years of experience and > 20 years of experience (*p* = 0.7), nor between initial (the first 2 of 6) vs. subsequent (the last 2 of 6) needle placements in the AR cohorts (AR-overlay *p* = 0.3, AR-plain *p* = 0.15).

**Table 1:** Placement accuracy

Cohort	Distance (mm) Mean ± SD
Fusion	4.5 ± 1.7
AR-overlay	7.0 ± 4.7
AR-overlay*	5.9 ± 2.6
AR-plain	4.7 ± 1.7
Freehand	9.2 ± 5.8

Fusion = ultrasound-based fusion; AR-overlay = goggle-based augmented reality system with stereoscopically projected anatomy; AR-plain = goggle-based augmented reality system without anatomical projections; Freehand = single, cognitive needle insertion without navigation system; SD = standard deviation

*Two data points, one from each of two different operators that represented outliers are excluded here

### Placement time

**Fig. 4:**
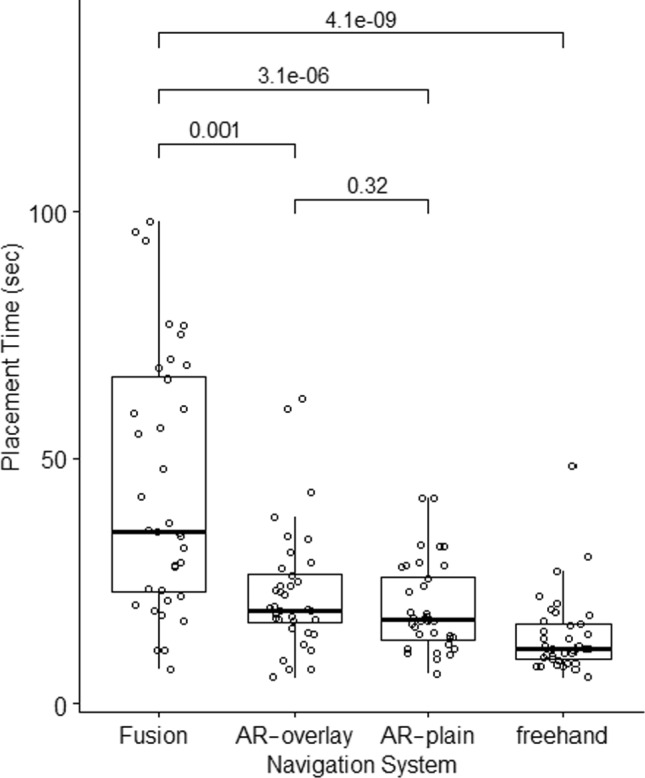
Needle placement time. Mean needle placement time, pooled for all six operators in each cohort. Fusion = ultrasound-based fusion, AR-overlay = goggle-based augmented reality system with stereoscopically projected anatomy, AR-plain = goggle-based augmented reality system without anatomical projections, Freehand = single, cognitive needle insertion without navigation system

Mean needle placement times using US-based fusion, AR-overlay, AR-plain, and freehand were 44.3 ± 27.7 s, 22.7 ± 12.6 s, 19.5 ± 8.9 s, 13.9 ± 8 s, respectively (Fig. 4, Table 2). After post hoc Bonferroni–Holm correction, needle placement time with US-based fusion was longer than with goggle AR, with or without anatomical overlay (*p* = 0.001, and *p* < 0.001, respectively). Freehand provided the fastest needle insertions compared to US-based fusion, AR-overlay, and AR-plain navigation systems with *p* < 0.001, *p* < 0.001, and *p* = 0.01, respectively. Placement times did not differ between AR-overlay and AR-plain, *p* = 0.32.

**Table 2:** Procedure times

Cohort	Placement time (min) Mean ± SD	Procedure time (min) Mean ± SD
Fusion	44.3 ± 27.7	19.5 ± 5.6
AR-overlay	22.7 ± 12.6	34.0 ± 10.2
AR-plain	19.5 ± 8.9	22.7 ± 8.6
Freehand	13.9 ± 8	14.8 ± 1.6

Fusion = ultrasound-based fusion; AR-overlay = goggle-based AR system with stereoscopically projected anatomy; AR-plain = goggle-based AR system without anatomical projections; Freehand = single, cognitive needle insertion without navigation system; SD = standard deviation

### Procedure time

**Fig. 5:**
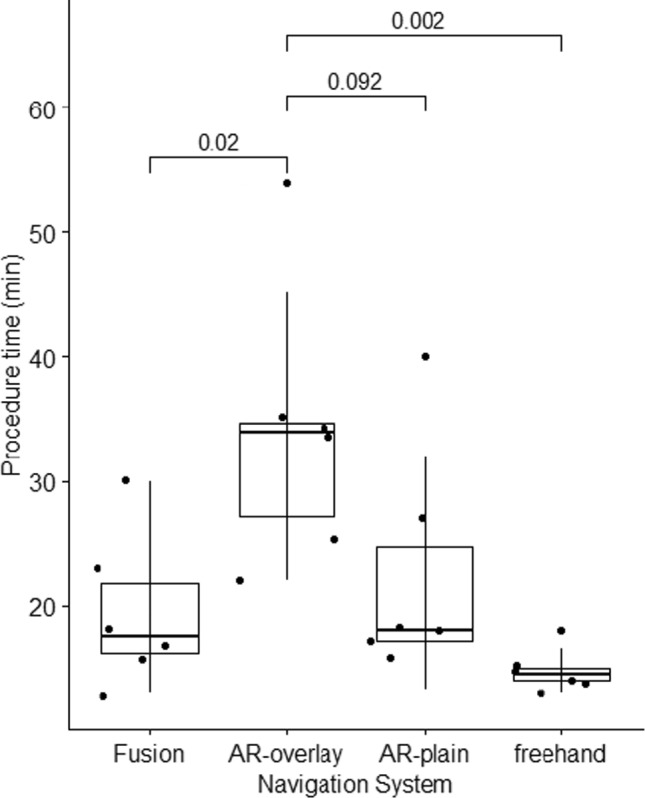
Procedure time. Overall procedure time for all six operators in each cohort. Fusion = ultrasound-based fusion, AR-overlay = goggle-based augmented reality system with stereoscopically projected anatomy, AR-plain = goggle-based augmented reality system without anatomical projections, Freehand = single, cognitive needle insertion without navigation system

Overall procedure times for US-based fusion, AR-overlay, AR-plain, and freehand were 19.5 ± 5.6 min, 34 ± 10.2 min, 22.7 ± 8.6 min, and 14.8 ± 1.6 min, respectively. Hence, the longest procedure times were registered with AR-overlay [compared to US-based fusion, freehand, and AR-plain, *p* = 0.02, *p* = 0.002, and *p* = 0.09, respectively (Fig. 5, Table 2)]. Procedure times with goggle AR were longer for operators with > 20 years’ experience compared to operators with ≤ 2 years’ experience (*p* = 0.02). Secondary time measurements are available in Online Resource 1 including Table E1.

## Discussion

In this phantom study, the goggle-based AR guidance system showed no difference in placement accuracy compared to a commercially available ultrasound-based fusion navigation platform and both navigation systems outperformed freehand insertion. However, small differences in placement accuracy and procedure times between the navigational systems became apparent with different display modes such as superimposition of the stereoscopically projected anatomy in the AR system (AR-overlay cohort). Moreover, the US-based fusion cohort required the longest single needle placement times but overall less procedure time compared to the goggle-based AR system.

The complexity of percutaneous needle insertions guided with US, CT or MRI depend on size, location and visibility of the target lesion, potential access routes, adjacent critical structures, and the experience of the operator [17]. Despite the importance of precise image-guided target localization to outcomes and reproducibility, interventional radiology is only slowly exploring the world of augmented guidance and navigation, having the fewest number of published studies among ten other procedural specialties [7]. Conventional US does not provide 3D visualization, while standard “step and shoot” CT guidance does not provide real-time needle position and leads to undesirable radiation exposure. Hence, the virtual fusion of real-world environments with stereoscopically projected, digital content may be useful to bridge the interaction between hands-on procedures and assistive, visual information, which is especially critical in complex biopsy or composite ablation settings [14]. Studies of AR navigation systems using smartphone or tablet devices have demonstrated placement accuracies < 5 mm and decreased procedure times compared to conventional CBCT techniques [8, 11, 13, 18, 19]. In contrast to 2D screen-based devices, smart goggles can provide an interactive 3D environment, which is directly superimposed over the patient. In recent studies, AR guidance with smart goggles also led to a reduction in procedure time and balanced variability among operators [8, 9, 19]. The current study evaluated performance and procedure times with a goggle-based AR guidance system compared to a commercial US-based fusion navigation system, restricted to no exposure to radiation. The AR-based system could simultaneously project the real-time US imaging and the needle guidance over the operational field with the option to include the stereoscopically projected anatomy, all in one display. With the display mode that included the anatomic model, the abundant occupation of the field of view partially blocked the view of the needle guidance, which may have contributed to slightly reduced placement accuracies and longer procedure times in this cohort. Without the dense anatomic overlay in the operational field, placement accuracy with AR showed no difference (< 5 mm) to the US-based fusion system, with comparable procedure times. The US-based fusion system required the longest placement times most likely because operators slowly and carefully advanced the needle attempting to maintain the center position in the bull’s-eye view.

The skin entry points and target paths were intentionally chosen to create out-of-plane target trajectories in ultrasound to simulate challenging needle insertions. Target and trajectory planning are often based on preprocedural CT in US-based fusion. With an ideal registration, the pre-calculated trajectory may be safely used to reach the target, even without the use of additional US, based on EM tracking of the needle and patient. An out-of-plane target approach with US is possible with the EM-tracked needle with both navigation systems. The ultrasound-based fusion system can define targets with preprocedural imaging or procedural CT, even when not visible on US. In the current goggle-based AR system, the target selection and trajectory planning were dependent on the visualization of the target on live ultrasound. Therefore, a lesion not visible on US cannot be targeted with the current AR system workflow, representing a major difference between the systems. Park et al. developed an AR-based navigation system that operates with smart goggles without the use of any live imaging modality but it also requires preprocedural segmentation of the anatomy to superimpose the stereoscopic projection over the subject [9]. The smart goggle AR system does not account for patient movement including respiratory motion with respect to the EM field generator. While the live US projection remains intact, registration to the CT anatomy data will suffer. Inaccurate registration with any navigation system can mislead the operator, leading to unintended placement errors. In this study, image registration became unstable when operating at the edge of working space, which led to inaccurate display and required reset. Tracking patient motion or breathing with an EM dynamic reference fiducial or respiratory gating may compensate or correct for patient motion or breathing and minimize registration error. Furthermore, the ultrasound can be displayed independently.

In both guidance systems, there was no difference in placement accuracy between interventional radiologists with ≤ 2 years or > 20 years of experience. Due to the small study number of operators with different experience levels, these results have to be interpreted with caution. However, reduced variability among physicians of different experience levels using AR systems has also been reported in the literature in larger cohorts [8, 9]. In this fashion, fusion and AR-based navigation systems may promote standardization and reproducibility across a variety of operators that otherwise require years of experience to achieve. Conventional approaches to navigation such as freehand depend upon cognitive processes and estimation of angles and geometries, which require experience, training, and time. The goggle-based AR system superimposes the live US image directly over the phantom body which improves visuospatial orientation in US, especially for less experienced users. Hence, navigation systems may decrease the learning curve and potentially enhance training and education [10, 20–22].

Due to ergonomics, complexity, and workflow for both AR and fusion, training for navigation systems required more time and technical assistance with initial system preparation, CT upload, and registration (AR more than US-based fusion). Segmentation of CT images and their upload was an additional required step in the AR system. However, it is contextually relevant to point out that the AR system is an early generation system, and likely to become streamlined and more functional, versus the US-based fusion platform whose workflow has matured since receiving marketing clearance by the US Food and Drug Administration in 2006 [23]. This study did not evaluate all methods of use for both systems.

This study has several limitations. The evaluation was conducted using a stationary phantom. Variables, such as patient movement, respiratory motion, and organ deformation are important factors that can affect navigational performance and clinical translation and were not evaluated in this study. Since both navigation systems were based on a preprocedural CT for registration, the images may not depict real-time patient anatomy and deformation during the procedure. Especially in the AR system, organ motion and tissue deformation are factors that could cause an inaccurate registration of static stereoscopic projections, reducing accuracy of interventions. All participants used the smart goggles AR system with a complete 3D anatomical overlay before operating without visual interference of the stereoscopically projected anatomy. This could have led to individual learning and training of the process and technique, affecting the accuracy between the two study cohorts. However, neither placement time, registration time, planning time nor overall procedure time differed with or without an anatomic superimposition between the two AR-goggle cohorts. There was also no difference in placement accuracy between initial placements vs. subsequent placements, which might have suggested a potential role of a learning effect. A new phantom, designed to “heal” and not show past needle trajectories on US was used by all operators for all sets of experiments. However, it is possible that needle trajectories from previous insertions may have aided or hindered subsequent needle insertions. The freehand cohort was unrealistically constrained to one needle insertion attempt, thus oversimplified in order to equalize radiation doses and to study “worse-case scenario” of single trajectories without intervening check scans. The sample size was small, however comparable to current phantom studies in literature. Finally, the study was conducted on an angiography table. Due to the proximity of the C-arm, the potential for EM interference with the EM tracking-based system performance cannot be ruled out.

In this phantom study, the goggle-based AR system showed comparable needle placement accuracy to the clinically validated US-based fusion platform and both systems outperformed freehand insertion. Small differences in placement accuracy and procedure times between the navigation systems were especially apparent with different display modes, with more challenging performance seen with registration and superimposition of the stereoscopically projected anatomy. Further assessment of AR technology and display modes under clinical scenarios of biopsy and ablation are warranted. AR enables a superimposition of real-time ultrasound imaging over the body with the live virtual needle trajectory, which may add helpful information to specific clinical settings, especially for inexperienced users. Deployment of such navigation technologies could enhance standardization, reproducibility, visuospatial orientation, and geometric estimations which are critical to needle-based biopsy and ablations.

## Supplementary Information

Below is the link to the electronic supplementary material.

Supplementary file1 (DOCX 18 kb)

## Abbreviations

CT: Computer tomography
CBCT: Cone-beam computer tomography
EM: Electromagnetic
US: Ultrasound
PET: Positron emission tomography
MRI: Magnetic resonance imaging
AR: Augmented reality
IR: Interventional radiology
AR-plain: Goggle-based augmented reality system without stereoscopically projected anatomy
AR-overlay: Goggle-based augmented reality system with stereoscopically projected anatomy
SD: Standard deviation
IQR: Interquartile range
