# Supplementary material for: Smart goggles augmented reality CT–US fusion compared to conventional fusion navigation for percutaneous needle insertion
Source: Int J Comput Assist Radiol Surg. 2024 May 30;20(1):107–15. doi: 10.1007/s11548-024-03148-5 (PMC11758159; doi:10.1007/s11548-024-03148-5)
Supplement: Supplementary file 1 — Supplementary file1 (DOCX 18 kb) [file 11548_2024_3148_MOESM1_ESM.docx]

Supplementary Information

**Title**

Smart Goggles Augmented Reality CT-US Fusion Compared to Conventional Fusion Navigation for Percutaneous Needle Insertion

**Journal**

IJCARS – International Journal of Computer Assisted Radiology and Surgery

**Authors**

Tabea Borde, M.D., Ph.D.^1,2^, Laetitia Saccenti, M.D.^1,3^, Ming Li, Ph.D.^1^, Nicole A. Varble, Ph.D.^1,4^, Lindsey A. Hazen, B.S.N.^1^, Michael T. Kassin, M.D.^1^, Ifechi N. Ukeh, M.D.^1^, Keith M. Horton, M.D.^5^, Jose F. Delgado, MS^1,6^, Charles Martin 3rd, M.D.^7^, Sheng Xu, Ph.D.^1^, William F. Pritchard, M.D., Ph.D.^1^, John W. Karanian, Ph.D.^1^, Bradford J. Wood, M.D.^1,6^

^1^ Center for Interventional Oncology, Radiology and Imaging Sciences, Clinical Center, National Institutes of Health, Bethesda, MD 20892, USA

^2^ Department of Diagnostic and Interventional Radiology, School of Medicine & Health, Technical University Munich, Munich 81675, Germany

^3^ Henri Mondor Biomedical Research Institute, Inserm U955, Team N°18, Créteil, France

^4^ Philips Healthcare, Cambridge, MA 02141, USA

^5^ Department of Radiology, Georgetown Medical School, Medstar Washington Hospital Center, Washington, DC 20007, USA

^6^ Fischell Department of Bioengineering, University of Maryland, College Park, MD 20742, USA

^7^ Department of Interventional Radiology, Cleveland Clinic, Cleveland, OH 44195, USA

Corresponding Author during submission and revision:

Tabea Borde, M.D., Ph.D.; Email: tabea.borde@nih.gov

Corresponding Author for published manuscript:

Bradford J. Wood, M.D.; Email: bwood@nih.gov

# Online Resource 1

## Secondary time endpoints

Secondary endpoints were system preparation time (time from turning on system to time of registration), planning time (for US-based fusion: time of selecting targets and entry points; for goggle AR and freehand: time from selecting a target to skin puncture), registration time (time for registration process), and training time (time to independently use system).

Since US-based fusion provides a fusion image of the uploaded CT and live ultrasound, targets were selected and needle trajectories were defined pre-procedurally. Intraprocedural target planning times for AR-overlay (16.8 ± 6.2 min) and AR-plain (13 ± 4.8 min) did not differ (*p* = 0.25). Freehand required the least target planning time (8 ± 1.2 min), less than for AR-overlay and AR-plain, p = 0.01 and p = 0.05, respectively. Freehand did not require any registration time. Within the guidance systems, goggle-based AR required the longest absolute registration times (AR-overlay 12 ± 11 min, AR-plain 6.2 ± 6 min, *p* = 0.27) compared to US-based fusion (1.3 ± 1.4 min, *p* = 0.04, *p* = 0.06, respectively). Training times for US-based fusion and AR-based navigation systems were not different (US-based fusion 18.5 ± 12 min, goggle AR 33 ± 22 min, *p* = 0.25), however absolute differences were noted between operators. Table E1 provides an overview of all procedure times. Only one operator had prior experience with the US-based fusion display that was used, however this could have biased results.

| **Table E1.** Procedure times for navigational devices | | | | | |
| --- | --- | --- | --- | --- | --- |
| Guide | Placement (sec)  Mean ± SD | Planning (min)  Mean ± SD | Procedure (min)  Mean ± SD | Registration (min)  Mean ± SD | Training (min)  Mean ± SD |
| Fusion | 44.3 ± 22.9 | 0 | 19.5 ± 6.1 | 1.3 ± 1.4 | 18.5 ± 11.6 |
| AR-overlay | 22.8 ± 5.2 | 16.8 ± 6.2 | 34 ± 11.2 | 12 ± 10.9 | 33.2 ± 22.4 |
| AR-plain | 19.5 ± 4.8 | 13 ± 4.8 | 22.7 ± 9.4 | 6.2 ± 5.6 |  |
| Freehand | 13.9 ± 4.3 | 8 ± 1.2 | 14.8 ± 1.7 | 0 | 0 |

Fusion = ultrasound-based fusion, AR-overlay = goggle-based augmented reality system with stereoscopically projected anatomy, AR-plain = goggle-based augmented reality system without anatomical projections, Freehand = single, cognitive needle insertion without navigation system, SD = standard deviation.
